# Hypoplastic acute myeloid leukemia in an elderly patient. A long‐term partial remission with low‐dose prednisone and G‐CSF

**DOI:** 10.1002/ccr3.2204

**Published:** 2019-05-17

**Authors:** Anwarul Islam

**Affiliations:** ^1^ Division of Hematology/Oncology, Department of Medicine Buffalo General Hospital Buffalo New York

**Keywords:** hypoplastic acute myeloid leukemia, prednisone, G‐CSF, filgrastim, myeloid malignancies, elderly patients

## Abstract

Hypoplastic acute myeloid leukemia (AML) is a rare variant of AML that mainly affects the elderly and accounts for 5%‐7% of de novo AML. Prognosis for this disease is poor, and there is no standard therapy. We have treated an elderly patient with hypoplastic AML with low‐dose prednisone and G‐CSF with good results. We propose that this treatment may be a viable alternative to supportive therapy alone, or the tenuous chemotherapeutic challenge to elderly patients with hypoplastic AML.

## 1 INTRODUCTION

We have used low‐dose prednisone in conjunction with Granulocyte‐coloney stimulating factor (G‐CSF) to treat an elderly patient with hypoplastic acute myeloid leukemia (AML). Our findings indicate that such treatment is safe and effective in the long‐term survival of the patient, is compatible with good health, and is devoid of toxicity.

While most cases of AML are usually hypercellular or at least normocellular, 5%‐12% of all cases of AML are of the hypocellular variety and typically called hypoplastic or hypocellular AML.^1^, ^2^, ^3^, ^4^, ^5^, ^6^ Hypoplastic AML is defined by bone marrow hypocellularity (cellularity <20% in trephine biopsy specimen, although in some earlier reports cellularity <40% or even 50% was considered to be hypocellular),^2^, ^4^, ^7^, ^8^ with increase of bone marrow blasts ≥20% and few or no blast cells in the circulating blood and peripheral blood leukopenia <4000/μL^9^ although pancytopenia is not uncommon.^2^, ^10^, ^11^ Patients with hypoplastic AML have higher median age (67.5 vs 44.0 years; *P* < 0.001) and lower survival rate (6.7% vs 26.7% at 5‐years; *P* = 0.035), respectively, in comparison with other AML variants.^9^ Conventionally, hypoplastic AML patients are treated with supportive care alone or with various combination regimens including an anthracycline, cytosine arabinoside, 6‐thioguanine, and vincristine^2^ to which they respond poorly or do not respond at all. Occasionally, complete remissions have been observed in patients with hypoplastic AML following administration of G‐CSF without antileukemic therapy.^12^, ^13^ These occasional reports refer to patients in whom an infection complicated the hypoplastic AML at diagnosis, and G‐CSF was given with the aim of increasing the neutrophil count to ameliorate the infection. There are no reports of the use of G‐CSF in conjunction with prednisone in the treatment of patients with hypoplastic AML.

We have treated a newly diagnosed elderly patient with hypoplastic AML with a combination of G‐CSF (filgrastim) and low‐dose prednisone. The rationale of the treatment was that this combination can induce differentiation of the immature myeloid cells, inhibit proliferation of the leukemic cells,^14^, ^15^, ^16^, ^17^ and potentially increase or at least stabilize the platelet count. This protocol improves the circulating neutrophil count and avoids the undesirable effects of chemotherapy.

Our patient demonstrated positive satisfactory results. She went into a stable continuous partial remission (reduction of 40% blasts to 20% blasts in the marrow). The circulating neutrophil count increased and was generally within normal limits, while the hemoglobin level was maintained at or around 8 g/dL with occasional blood transfusion. The platelet count was low but remained stable (around 35 000/μL) and needed occasional platelet transfusion. Petechiae and bruises did not appear. Occasional minor elevations of the neutrophil count (up to 15 000 μL) were treated with diminished allocations of G‐CSF. Blast cells were not identified in the peripheral blood at any time.

The patient's response was prompt and was sustained over the long term. At present, she continues to do well >18 months postdiagnosis and initiation of therapy. She is relatively active and in good health. She sustained successfully for the treatment for severe herpes zoster and sciatica.

## 2 CASE REPORT

The patient, a 91‐year‐old white woman with a past medical history significant for hypertension, coronary arterial disease, aortic stenosis, arthritis, and atrial fibrillation presented at the emergency room per advice of her cardiologist who found her to be pancytopenic. On physical examination, the patient was noted to be anemic with mild shortness of breath but not in acute distress. She was mildly jaundiced, but there was no cyanosis or edema. Her abdomen was soft and nontender. Bowel sounds were heard. Liver, spleen, and kidneys were not palpable. There was no palpable lymphadenopathy. Heart sounds S1 and S2 were identifiable along with a soft ejection systolic murmur. The chest was clear to auscultation. Her vital signs were stable—blood pressure 125/70 mm Hg, pulse 72 pm, respiration 18 pm, and temperature 97.6°F. The family history was noncontributory.

Laboratory investigations revealed a WBC 1.0 × 10^9^/L, hemoglobin 6.6 g/dL with raised MCV at 123.0 fL, MCH 41.0 pg, and a platelet count of 24 × 10^9^/L (Table 1). A manual differential of her peripheral blood smear revealed 38.4% neutrophils and 55.8% lymphocytes, 2.9% monocytes, 2.9% eosinophils, and no blast cells. Her serum iron level was elevated at 171 µg/dL (normal range 20‐115), the iron binding capacity was slightly low at 242 µg (normal range 250‐450), and the per cent saturation was slightly high at 71% (normal range 20‐55). Her ferritin was raised to 300 ng/mL (normal range 15‐250). Her reticulocyte count was within normal limits 2.2%. The LDH (247 units/L), B12 (306 pg/mL), and folic acid (15.2 ng/mL) levels were also within normal limits. Her complete metabolic profile was mostly normal except for bilirubin, which was slightly increased at 1.9 mg/dL. The routine urine analysis was negative. The patient received two units of packed RBC with 20 mg of furosemide intravenously before the second unit at the time of admission.

**Table 1:** Hematological parameters at diagnosis and at subsequent intervals

Time	Hg g/dL	WBC 10^9^/L	Platelet 10^9^/L
Diagnosis	6.6	1.0	24
2 mo	8.7^a^	1.8	30
4 mo	8.7	1.7	31
6 mo	10.3^a^	3.5	53^a^
8 mo	10.1	5.1	72^a^
10 mo	10.1	6.6	67
12 mo	9.3	15.5	46
14 mo	9.3	9.8	30
16 mo	8.8	3.3	28
18 mo	8.7	12.1	23

a Status post–blood and platelet transfusion.

**Figure 1:**
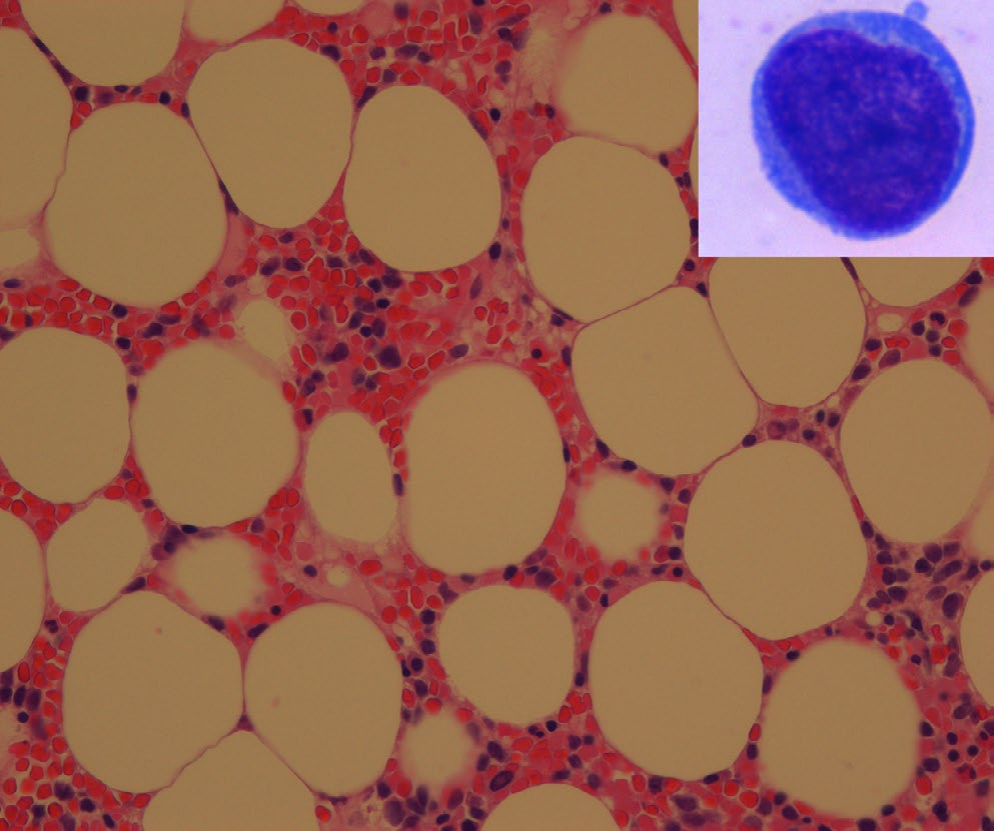
Bone marrow trephine biopsy specimen obtained at diagnosis showing hypocellular marrow. Cellularity around 10%. The inset shows a blast cell

A bone marrow aspirate, clot sections, and a trephine biopsy specimen revealed a diagnosis of hypoplastic AML—blasts 40% in the marrow with cellularity 10% per trephine biopsy specimen (Figure 1). Plasma cells were mildly increased and constituted about 10% of the marrow cells.

**Figure 2:**
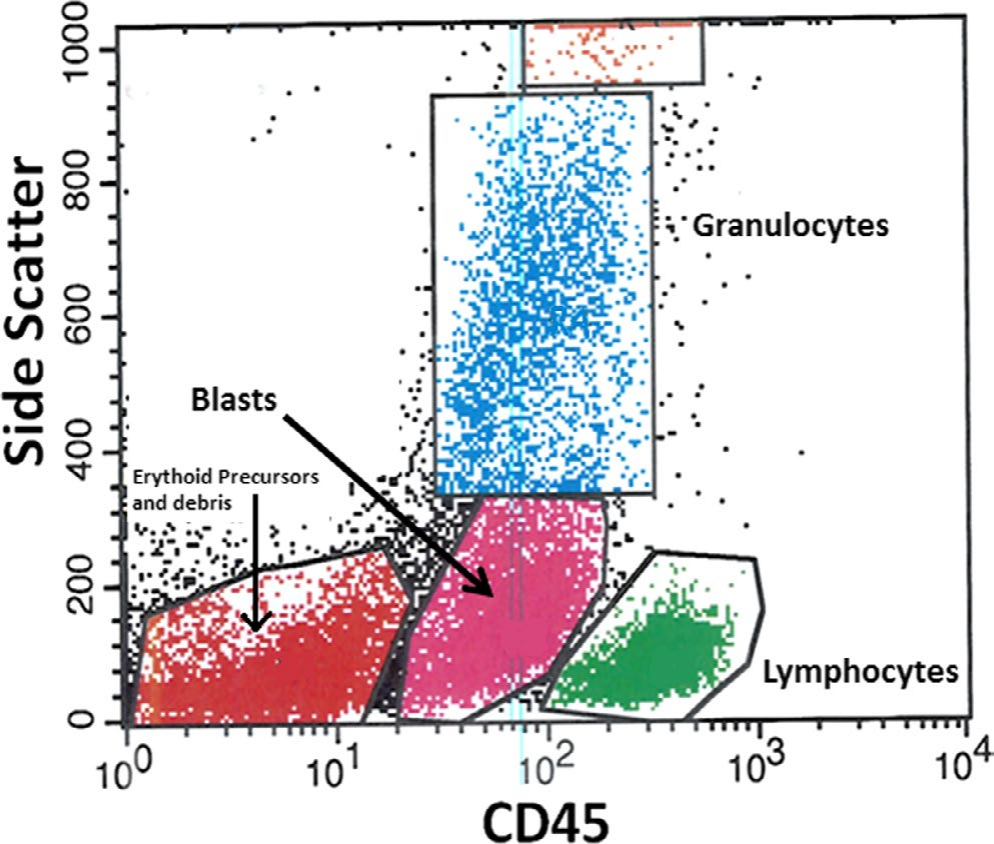
Flow cytometry results of bone marrow aspirate obtained at diagnosis: cluster analysis of blasts using CD45/SSC. Pink: blasts; green: lymphocytes; blue: granulocytes; and red: erythroid precursors and debris

Flow cytometry studies (Figure 2) of the bone marrow aspirate revealed an increased (20%) population of immature cells (blasts), as evidenced by HLA‐DR and CD34 expressions. This blast population expressed dim CD45 with minimal side scatter. By forward scatter, these cells were medium to large sized, and they expressed CD34, CD117, CD13, CD14, CD38, HLA‐DR, and were negative for CD3, CD20, CD11_c_, CD15, and CD64. In addition, a population of cells (1% of total marrow cells), medium to large size (by forward scatter) expressing bright CD38, CD56, CD138, consistent with abnormal plasma cells, were also noted. Cytogenetic studies: A total of 20 metaphase spreads were analyzed by G‐banding, which revealed a normal female karyotype of 46, XX. No apparent clonal chromosomal aberrations were identified.

Because of her age (91 years) and a diagnosis of hypoplastic AML, the patient was referred to Hospice care. However, the patient's family declined this option and requested treatment that did not involve chemotherapy. It was at this point that the patient was started on low‐dose prednisone 20 mg orally daily, pantoprazole 20 mg/d, and G‐CSF (filgrastim) 300 µg subcutaneously three times (Monday, Wednesday, and Friday) a week. The patient did not receive concomitant administration of any other cytokines or any chemotherapy. Prednisone was gradually tapered and has been reduced to 5 mg orally daily for the last 5 months.

Within 2 weeks, her circulating neutrophil count began to increase. The hemoglobin concentration and platelet count remained low and required RBC and platelet transfusions from time to time but the patient remained clinically and hematologically stable.

Six months following the initiation of the treatment, her WBC count rose to 3.5 × 10^9^/L, hemoglobin rose to 10.3 g/dL (with an occasional blood transfusion), the MCV remained high at 110.8 fL, and the platelet count attained 53 × 10^9^/L with an occasional platelet transfusion (Table 1).

Nine months following the start of the treatment, her WBC count was normal at 4.7 × 10^9^/L, the hemoglobin was 10.0 g/dL, the MCV remained high at 121.0 fL, and the platelet count increased to 78 × 10^9^/L.

At 12 months of treatment, the WBC count was elevated to 15.5 × 10^9^/L (at which time the filgrastim dose was lowered to 300 µg twice a week), hemoglobin level was 9.3 g/dL, and the platelet count was 46 × 10^9^/L (Table 1).

Sixteen months following the start of the treatment, her WBC count fell to 3.3 × 10^9^/L (and the filgrastim dose was raised to 300 µg three times a week), the hemoglobin was 8.8 g/dL, the MCV remained unchanged, and the platelet count receded to 28 × 10^9^/L (Table 1). The patient remained stable and in relative good health.

**Figure 3:**
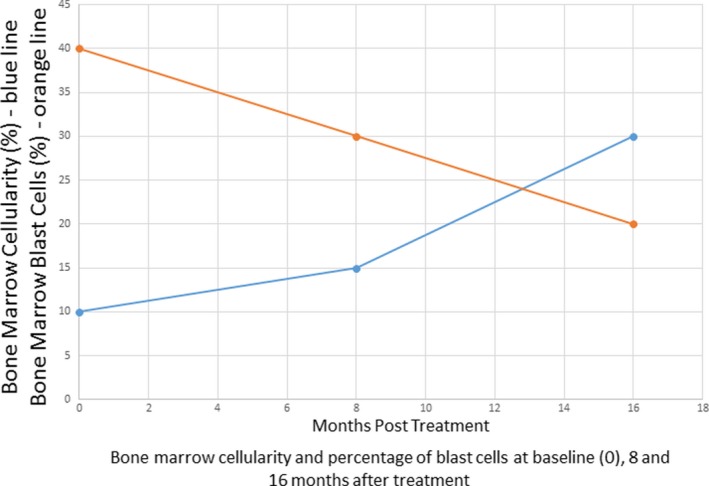
The graph shows percentage of bone marrow cellularity and percentage of blast cells at diagnosis and at 8 and 16 mo after treatment

A BM aspiration and biopsy conducted about 8 months following the start of G‐CSF and low‐dose prednisone therapy revealed persistent hypoplastic AML—30% blasts in the marrow and 15% cellularity in the trephine biopsy specimen (Figure 3).

**Figure 4:**
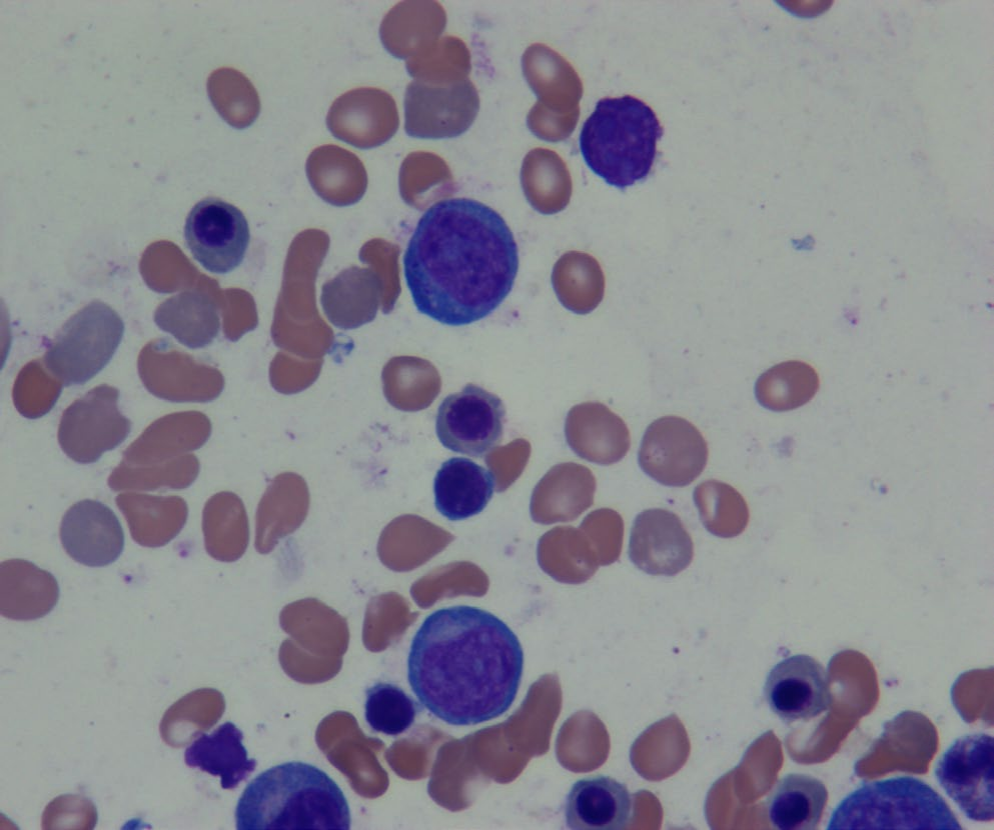
Bone marrow aspirate obtained 16 mo following the start of treatment showing immature myeloid precursor cells

**Figure 5:**
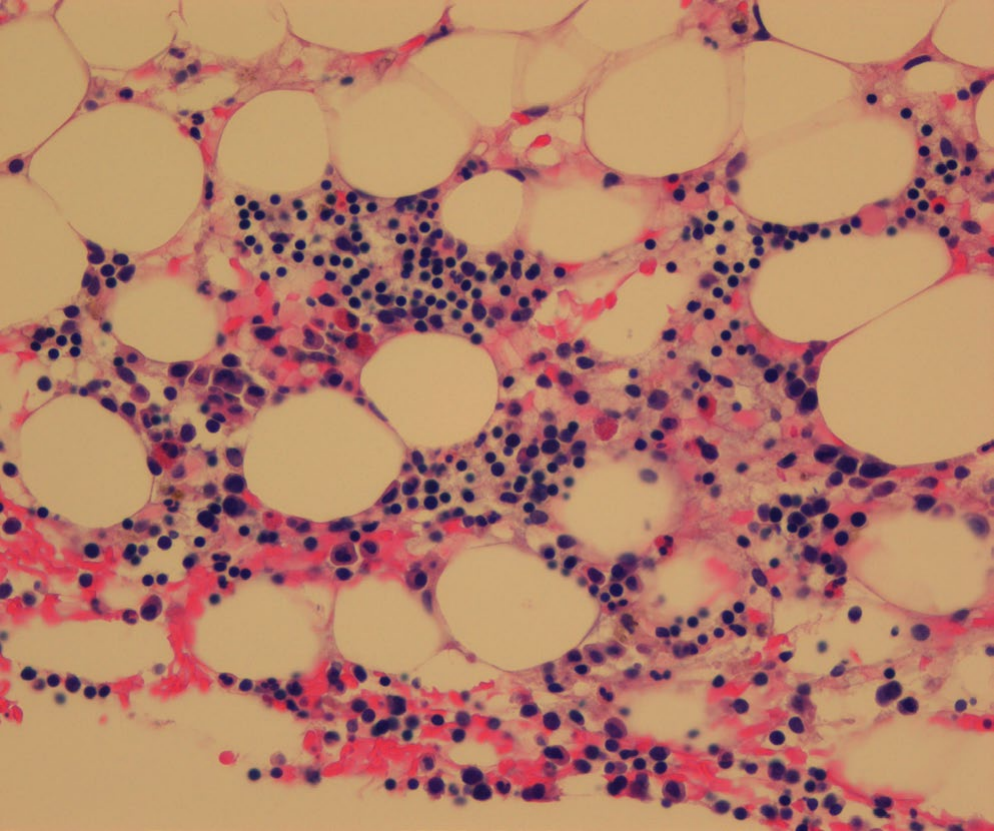
Bone marrow trephine biopsy specimen obtained 16 mo following the start of treatment showing hypocellular marrow but with improved marrow cellularity around 30%

**Figure 6:**
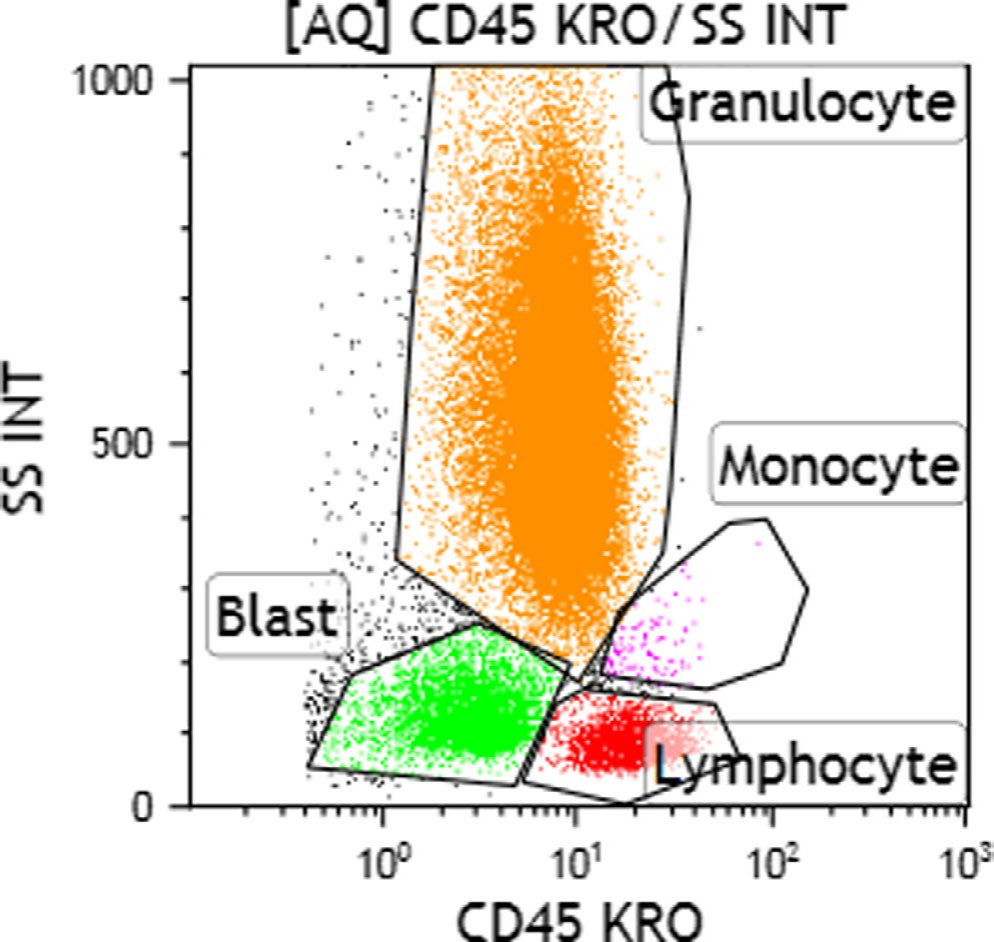
Flow cytometry results of bone marrow aspirate obtained at 16 mo of treatment: cluster analysis of blasts using CD45/SSC. Green: blasts; pink: monocytes; red: lymphocytes; and orange: granulocytes

A BM aspiration and biopsy performed at 16 months following the start of the protocol once again showed persistent hypoplastic AML with a 20% blast cell population in the marrow (Figures 3 and 4) and 30% cellularity per trephine biopsy specimen (Figures 3 and 5). Flow cytometry findings (Figure 6) at this time were equivalent to the flow cytometric studies performed at diagnosis. Cytogenetic studies: A total of 20 metaphase spreads were analyzed by G‐banding, which revealed a normal female karyotype of 46, XX. No apparent clonal chromosomal aberrations were identified. Molecular studies (IDH1, IDH2, FLT3, NPM1, and TP53) were negative.

The patient thus showed a good response to G‐CSF and low‐dose prednisone therapy particularly with respect to the peripheral neutrophil count, a reduced blast cell population in the marrow, and an improved bone marrow cellularity. Although the patient continues to show the presence of a greater than normal number of blast cells in the marrow and remains mildly anemic as well as thrombocytopenic, she continues to remain clinically stable without requiring blood or platelet transfusion or antibiotic therapy.

## 3 DISCUSSION

Hemopoietic growth factors such as G‐CSF are widely used in hemopoietic and nonhemopoietic malignancies to reduce the duration of neutropenia induced by chemotherapy and its infectious complications. In AML, G‐CSF and GM‐CSF have been used to reduce the duration of neutropenia after induction and consolidation therapy. The benefit of such treatment strategies remains controversial. However, most studies report a shorter duration of neutropenia with a lower incidence of infections in the hemopoietic growth factor–treated patients.^18^, ^19^, ^20^ It is believed that hematopoietic growth factors when administered concomitantly with chemotherapy this treatment induces a recycling of the leukemic cells and increases their chemosensitivity.^18^ In some studies, patients treated with G‐CSF showed statistically higher rate of complete remission with improvement of disease‐free survival, but not overall survival.^21^ These studies have thus demonstrated that G‐CSF may have an antileukemic effect^22^ or may contribute to the antileukemic effect of the chemotherapy.^21^ It has been shown that the leukemic cells display hemopoietic growth factor receptors,^23^ and some authors have shown that G‐CSF also induces differentiation and apoptosis of leukemic cells.^24^ Complete remission of hypoplastic AML has been reported with G‐CSF alone without chemotherapy.^22^

Steroid drugs, such as prednisone on the other hand, improve or at least stabilize the platelet count by slowing the body's response to disease and lowering the activity of the immune system. It has also been suggested that prednisone improves platelet counts by suppressing systemic reticuloendothelial phagocytic function.^25^, ^26^ Although glucocorticoids have been used for many years for their immunosuppressive, anti‐inflammatory, and cytotoxic effects, but their precise mechanism of action has not yet been fully elucidated. It has been reported that glucocorticoids induce apoptosis in leukemic cells through binding to glucocorticoids receptor and to transcription regulators, such as NF‐kB, AP‐1, and oncogene *c‐myc*, thus supporting their use as chemotherapeutic agents for leukemias, lymphomas, and myeloma .^27^, ^28^, ^29^

Hypoplastic AML is indolent, bears relatively low tumor cell burden, and usually pursues a less aggressive course than standard AML.^2^ Conservative treatment using prednisone and G‐CSF may offset the possible increased morbidity and mortality from intense chemotherapy. Patients with hypoplastic AML are usually elderly, have comorbidities, may have life‐threatening infections associated with the malignancy, and are unlikely candidates for chemotherapy. In such circumstances, treatment with low‐dose prednisone and G‐CSF may be advantageous because the side effects are rare, the quality of life is improved, the patients may require less hospitalization, and outpatient care is made more feasible.

G‐CSF is known as a cytokine which induces differentiation and proliferation of granulocyte progenitor cells resulting in an increased number of peripheral neutrophils.^23^, ^30^ Infection‐related mortality is particularly high among these patients during the period of neutropenia. In this situation, the administration of G‐CSF may play a significant role in improving the neutrophil count, shortening the neutropenic period, and reducing life‐threatening infections.

We believe the patient reported here is the oldest of patients with hypoplastic AML thus far reported in the literature. Because of her age and the diagnosis of hypoplastic AML, the patient was not considered an ideal candidate for chemotherapy and was referred to Hospice care. However, the patient's family declined this option and the patient then was started on low‐dose prednisone and G‐CSF therapy. Fortunately, the patient is doing well on this treatment requiring only occasional adjustments of the dose of G‐CSF 18 months after her initial diagnosis. The patient did have an episode of severe herpes zoster infection (involving the lumbar dermatome) to which she responded well with antiviral therapy and has recovered fully. It appears from our study that low‐dose prednisone and G‐CSF is safe in hypoplastic AML, did not stimulate the regrowth of leukemic cells but accelerated neutrophil recovery and thereby reduced the incidence of infection. The platelet count was maintained at a level which was sufficient to keep her free of bruising or bleeding without the requirement of platelet or blood transfusions.

In conclusion, low‐dose oral prednisone therapy and G‐CSF injections may represent a treatment options for hypoplastic AML in the elderly. The mechanism is unknown but possibly involves immunomodulatory actions of steroid (prednisone) and cycling of blast cell population into maturation and differentiation by G‐CSF.

## CONFLICT OF INTEREST

None declared.

## AUTHOR CONTRIBUTIONS

AI treated the patient and wrote the manuscript.
